# Letter from Paris

**Published:** 1843-03-18

**Authors:** 


### FOREIGN CORRESPONDENCE.

LETTER II.

[We publish the following extract, descriptive of an interesting affection, from the letter of a correspondent in Paris.]

* * * * * Dr. Labone, interne to M. Jobert, at St. Louis, has lately published some account of a disease of the uterus, to which M. J. has given the name of *dropsy of the neck*, and which he believes has not yet been described by authors. M. J. has, during the present season, frequently called the attention of the class to this form of affection. It occurs generally in lymphatic women, who have never borne children, and whose menstruation has been irregular and scanty. Without affecting materially the general health, this affection sometimes produces sympathetic phenomena, which may induce the suspicion of some serious disease of the uterus. The symptoms are only determined by compression. On examination, the os tincæ is hardly visible; the neck is larger than natural, and uniformly developed as in hypertrophy. At an early stage of the malady, a little prominence is visible at the orifice of the neck, but this disappears with the increase of volume of the neck which ensues. The surface of the neck is natural, except in the colour of the mucous membrane, which is rather pale, as if the blood had been pressed out. Where ulcerations exist, they are to be regarded as complications. On touching the neck of the womb, you do not find it hard, as its aspect would lead you to believe. The tissue offers but little resistance, and gives the feeling of fluctuation. An examination by the rectum enables you to judge less well of the consistence of the neck, but you readily discover its abnormal developement. If there be no ulceration, there is no purulent discharge. If you cautiously introduce a female catheter into the uterine orifice, a large quantity of a white or yellowish, clear, stringy liquid escapes, and the neck diminishes immediately in size. Sometimes this is discharged spontaneously, and instant relief is afforded.

The phenomena which this accumulation of fluid causes can be readily imagined. All the symptoms of hypertrophy of the uterus sometimes occur; and the disease is often confounded with it. A woman in M. Jobert's wards, suffering from this affection, had, previously to her entrance, been several months ineffectually subjected to treatment, for a supposed hypertrophy. It may be distinguished from hypertrophy,—1st. By the presence of the little tubercle which exists at the orifice of the neck, until it has acquired considerable developement. 2d. By the sense of fluctuation it gives on touching it. 3d. By the flow of liquid following the introduction of a catheter. The flow of the menses brings invariable relief. This fact is readily explained. The menstrual fluid, in flowing, necessarily carries with it the whole, or a portion of the liquid accumulated in the uterine cavity. M. Jobert thinks that the cause of the affection may be attributed to the anormal distention and increased secretions of the follicles we encounter about the neck of the uterus, and which become dilated in the same manner as the sebaceous follicles of the skin.

